# Does operative fixation affect outcomes of displaced medial epicondyle fractures?

**DOI:** 10.1007/s11832-016-0757-1

**Published:** 2016-07-08

**Authors:** Matthew Stepanovich, Tracey P. Bastrom, John Munch, Joanna H. Roocroft, Eric W. Edmonds, Andrew T. Pennock

**Affiliations:** 1 Naval Medical Center Portsmouth, Portsmouth, VA USA; 2 Pediatric Orthopedic and Scoliosis Center, Rady Children’s Hospital San Diego, 3030 Children’s Way, Suite 410, San Diego, CA 92123 USA; 3 Department of Orthopedic Surgery, University of California, San Diego, San Diego, CA USA

**Keywords:** Medial epicondyle fractures, Outcomes, Nonoperative, Operative

## Abstract

**Purpose:**

Long-term functional results remain equivocal between operative fixation and closed management of displaced humeral medial epicondyle fractures. The purpose of this study was to determine whether a functional difference exists between treatment types.

**Methods:**

One hundred and forty patients with a displaced medial epicondyle fracture between 2007 and 2014 met the inclusion criteria. Of this large cohort, only 12 patients agreed to return to clinic at a mean follow-up of 3 years for prospective evaluation. Data collection included radiographs, physical examination, validated outcome tools, and grip strength testing with a Jamar dynamometer.

**Results:**

Both groups were comparable with regard to age, dominant side injured, length of follow-up, preinjury sports involvement, and initial displacement (10 mm operative vs. 9 mm nonoperative); however, half of the surgical group presented with an associated unreduced elbow dislocation versus 0 % in the nonoperative group. Both treatment methods resulted in high patient satisfaction and elbow function scores. There were four osseous nonunions (67 %) and one malunion (17 %) in the nonoperative group versus none in the operative group (*p* = 0.015). Patients treated nonoperatively had a nonsignificant decrease in grip strength (9 ± 6 lbs) as compared to operative patients (6 ± 5 lbs, medium effect size eta = 0.25, *p* = 0.25).

**Conclusions:**

In this small cohort, operative management of displaced medial epicondyle fractures resulted in a higher rate of fracture union and return to sports. Other objective and subjective measures were similar between the two treatment groups.

## Introduction

Fractures of the humeral medial epicondyle were first described by Benjamin Granger in 1818 and account for up to 20 % of all pediatric elbow fractures, with a high rate occurring in conjunction with an elbow dislocation [1, 2]. Absolute indications for operative treatment remain limited to open fractures and nonreducible incarcerated fracture fragments. Relative operative indications include ulnar nerve dysfunction, elbow instability, high-level upper extremity athletes, and displaced fractures [3].

Medial epicondyle fracture displacement has been more closely evaluated recently, as fracture displacement is commonly cited as a relative surgical indication. Pappas et al., in 2010, demonstrated that, with standard AP elbow radiographs, the interobserver reliability of measuring fracture displacement is low and substantially declines when lateral radiographs are analyzed [4]. Further difficulty in utilizing radiographic displacement as a surgical indication was identified in 2010 when computed tomography (CT) analysis of apparent nondisplaced fractures were shown to have true displacement of up to 10 mm [5]. Even with these noted limitations, radiographic displacement continues to be utilized in most treatment algorithms. Numerous studies have reported equivocal long-term functional results between operative fixation and closed management of displaced humeral medial epicondyle fractures [6–8]. Even with these reported good results with nonoperative management, the reported rates of elbow stiffness, ulnar neuropathy, valgus instability, and fracture nonunion raise concerns [6–10]. Furthermore, recent computer simulation suggests that grip strength may be affected by malunion [11].

There remains a paucity of literature with direct comparison of nonoperative and operative treatment of displaced medial humeral epicondyle fractures. The purpose of the present study was to determine whether a functional difference exists between nonoperative and operative treatment of displaced medial humeral epicondyle fractures. We hypothesized that the nonoperative treatment group would have no functional differences on examination or with validated outcome measures compared to the operative treatment group, but would have a substantially higher rate of radiographic nonunion.

## Materials and methods

Institutional review board approval was obtained (IRB #141119). The electronic billing records of our institution were evaluated to identify fractures of the medial humeral epicondyle, including all surgical cases and clinic visits between 2007 and 2014. Patients were eligible for inclusion in the study if they sustained a closed medial humeral epicondyle fracture, had no evidence of epicondylar fragmentation, and had radiographic displacement of >2 mm on any view. Patients were excluded if they lacked injury- or treatment-associated anteroposterior and lateral radiographs, sustained a simultaneous ipsilateral upper extremity fracture, had an open fracture or an incarcerated fracture fragment, had intra-articular/condylar extension, had an unreducible elbow dislocation or subsequent loss of reduction of the ulnohumeral joint on radiographic follow-up, had associated ulnar nerve symptoms recorded at presentation, or a history of prior elbow surgery or deformity. While the decision to undergo operative or nonoperative management was made on an individual basis between the treating surgeon, the patient, and the family, factors such as timing of presentation, higher energy mechanism of injury, or fractures associated with dislocations increased the likelihood for surgical management.

Nonoperative management included long arm cast immobilization with the arm in neutral rotation in approximately 90° flexion for 3–4 weeks. Patients were subsequently provided instructions on daily range of motion exercises while continuing activity modifications. If elbow stiffness persisted at follow-up, formal physical therapy was initiated. Surgical treatment consisted of open reduction and internal fixation (ORIF) with partially threaded cannulated screw fixation with or without a washer. Postoperative long arm cast immobilization in neutral rotation in approximately 90° flexion was used for 1–3 weeks followed by range of motion exercises and formal therapy if motion limitations remained at subsequent follow-up. The exact method of immobilization, position of immobilization, and duration were staff-dependent. Implant removal was not routine and was performed on an individual basis.

The billing records initially identified 249 patients. Upon review, 140 patients (35 nonoperative and 105 operative) met the inclusion criteria. All eligible patients were contacted by letter and follow-up telephone call. All participants were offered a $25 gift card for participation in the study. Of the 140 eligible patients, 12 of these were reachable and agreed to return to clinic at a mean follow-up of 3 years for prospective evaluation.

After obtaining written consent, the patient and legal guardian (if the patient was under 18 years of age) completed a written questionnaire. The obtained questionnaire history included age at time of injury, current age, hand dominance, treatment method, length of immobilization, need for formal physical therapy, pre- and postinjury athletic level (specifically inquiring about participation in overhead sports), subsequent treatment including further surgeries, current symptoms, and treatment satisfaction. Prospective objective data collection included radiographs (standard anteroposterior and lateral radiographs, as well as the axial distal humerus view) [11], physical examination, and grip strength testing with a Jamar dynamometer. Physical examination motion and alignment measurements were all recorded using a goniometer. Stability testing was completed by performing standard varus and valgus stress examination. In addition, each patient underwent bilateral medial ulnar collateral ligament assessment with static and dynamic milking maneuver examination. Validated outcome measures were collected on each patient, including the shortened Disabilities of the Arm, Shoulder and Hand (QuickDASH) score, the Mayo Elbow Score, and the visual analog scale.

All elbow radiographs from time of initial injury to final follow-up were analyzed. Initial radiographs were analyzed for displacement using digital radiographs and the ruler tool on the PACS system (Merge PACS, Merge Healthcare Incorporated, 2013). Maximum displacement noted on any radiographic view was recorded. Final follow-up radiographs were assessed for the presence of implant retention, implant failure, malunion, or osseous nonunion.

### Statistical analysis

Descriptive statistics were calculated for all variables collected. Due to the small sample size, minimal probability statistics were performed. Nonparametric statistics were utilized for two primary outcome variables of grip strength (Kruskal–Wallis) and healing status (nonunion/malunion versus uneventful healing, Fisher’s exact test). SPSS version 12 was utilized for statistical analyses and alpha was set at *p* < 0.05 (SPSS, Inc., Chicago, IL).

## Results

Of the 12 patients that returned to clinic, six had been treated operatively and six had been treated by nonoperative methods. The operative and nonoperative groups were comparable with regard to age, dominant side injured, length of follow-up, and initial displacement (10 mm operative vs. 9 mm nonoperative); however, half of the surgical group had an associated elbow dislocation versus none in the nonoperative group (Table 1). Preinjury athletic involvement, as demonstrated by the percentage who competed in overhead sports, was not significantly different between the two groups (33 % in the nonoperative group compared to 50 % in the operative group). Utilizing data from our billing department, the approximate cost associated with the nonoperative treatment of a medial epicondyle fracture was $435, compared to $3492 for surgical treatment.

**Table 1:** Patient demographics

	Operative	Nonoperative
Age at treatment (years) (mean, range)	12 (11–13)	13 (8–16)
Length of f/u (years) (mean, range)	3 (1.5–6)	3 (1.5–4)
Initial injury displacement (mm)	10 ± 2	9 ± 6
Dislocation	50 %	0 %
Side injured		
Right	33 %	33 %
Left	67 %	67 %

At final follow-up, the range of motion was similar between the two groups. There was a slightly increased cubitus valgus alignment when comparing the nonoperative group (3.83°) to the operative group (0.67°) when the injured extremity was compared to the contralateral elbow (Table 2). A larger proportion of the operative group (67 %) had tenderness to palpation over the medial epicondyle than the nonoperative group (17 %). In each group, two of the six patients had mild increased laxity on stress examination when compared to the contralateral elbow. None of the patients reported clinical elbow instability on the questionnaire. Patients treated nonoperatively had a slight decrease in grip strength (9 ± 6 lbs) as compared to operative patients (6 ± 5 lbs, medium effect size eta = 0.25, *p* = 0.25).

**Table 2:** Clinical and radiographic outcomes

	Operative	Nonoperative
Nonunions (%)	0	50
Additional surgery (%)	0	0
Tenderness to palpation (%)	67	17
Elbow laxity to valgus stress (%)	33	33
Milking maneuver (%)	0	0
Elbow flexion test (%)	0	0
Tinel (%)	0	0
Wrist flexion strength (% with 5)	83	100
Wrist pronation strength (% with 5)	100	100
Elbow extension of injured extremity (°) (mean ± SD)	−5 ± 10	−9 ± 13
Difference in elbow extension (°) (mean ± SD)	1 ± 5	1.5 ± 11
Elbow flexion of injured extremity (°) (mean ± SD)	150 ± 5	144 ± 9
Difference in elbow flexion (°) (mean ± SD)	−1 ± 2	−1 ± 1
Wrist supination of injured extremity (°) (mean ± SD)	93 ± 3	94 ± 8
Difference in wrist supination (°) (mean ± SD)	0 ± 0	0 ± 0
Wrist pronation of injured extremity (°) (mean ± SD)	86 ± 5	88 ± 3
Difference in wrist pronation (°) (mean ± SD)	2 ± 4	0 ± 0
Grip strength of injured extremity (lbs) (mean ± SD)	56 ± 11	57 ± 19
Difference in grip strength (lbs) (mean ± SD)	6 ± 5	9 ± 6
Elbow coronal alignment of injured extremity (°) (mean ± SD)	14 ± 3	14 ± 5
Difference in elbow coronal alignment (°) (mean ± SD)	1 ± 1	4 ± 6

**Fig. 1:**
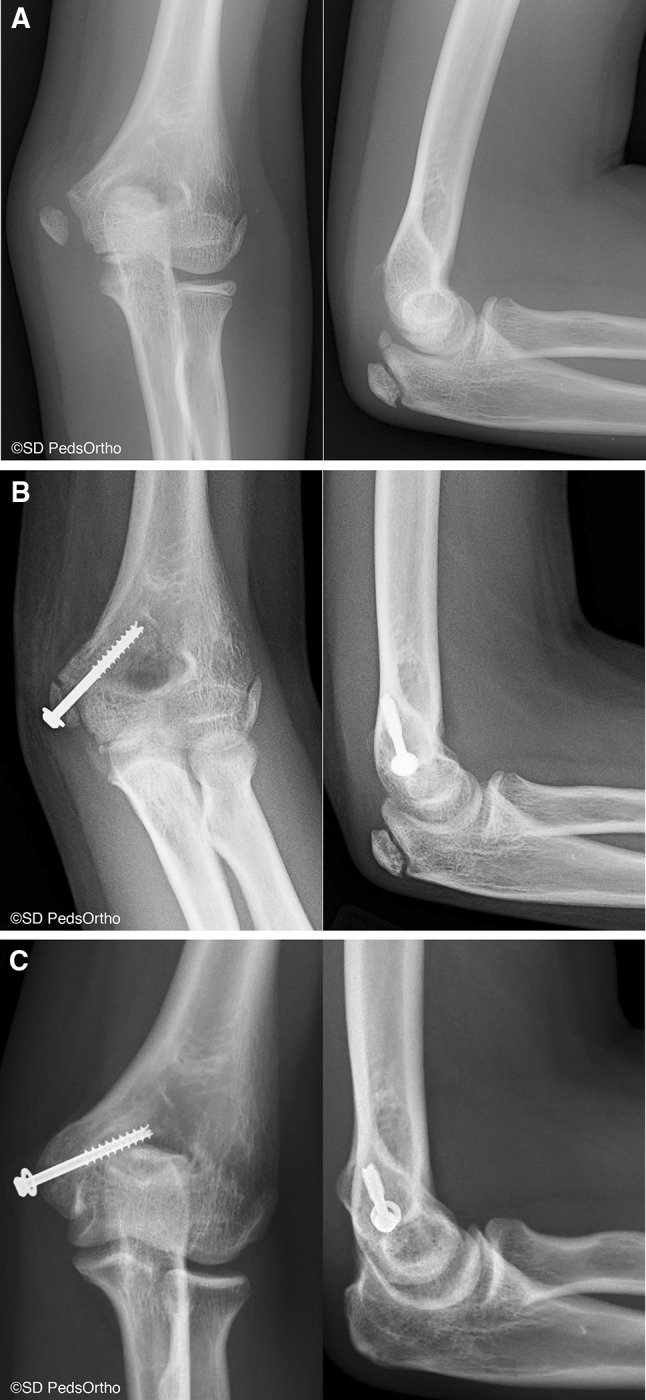
**a** Anteroposterior and lateral radiographs of a 12-year-old female gymnast who sustained a displaced medial epicondyle fracture. **b** Postoperative images. **c** Radiographs obtained 3.25 years postoperatively demonstrating a healed fracture with mild hardware prominence and irritation

Both treatment methods resulted in high patient satisfaction and elbow function scores (Table 3). There were four osseous nonunions (67 %) and one malunion (17 %) in the nonoperative group versus none in the operative group (*p* = 0.015). Two of the six nonoperative patients did not return to sports, whereas all operative patients returned to full sporting activities. One patient did not participate in sports preinjury and has decided not to pursue them postinjury. The other patient was a 13-year-old gymnast with a nonunion who decided to transition to diving after her injury. All operative patients retained their hardware without radiographic evidence of screw breakage/implant failure. One operative patient did have very mild hardware prominence, but this was asymptomatic unless directly palpated and the patient and family did not desire removal (Fig. 1a–c).

**Table 3:** Subjective outcomes

	Operative	Nonoperative
Full return to sports	100 %	67 %
Pain score (0 = no pain)	0 (all 0)	0 (all 0)
Satisfaction score (10 = fully satisfied)	9.8 (range 9–10)	10 (all 10)
DASH score (0 = no disability)	2.1 (range 0–6)	1.2 (range 0–6)
Mayo Elbow Score (100 = perfect)	100 (all 100)	100 range (all 100)

## Discussion

**Fig. 2:**
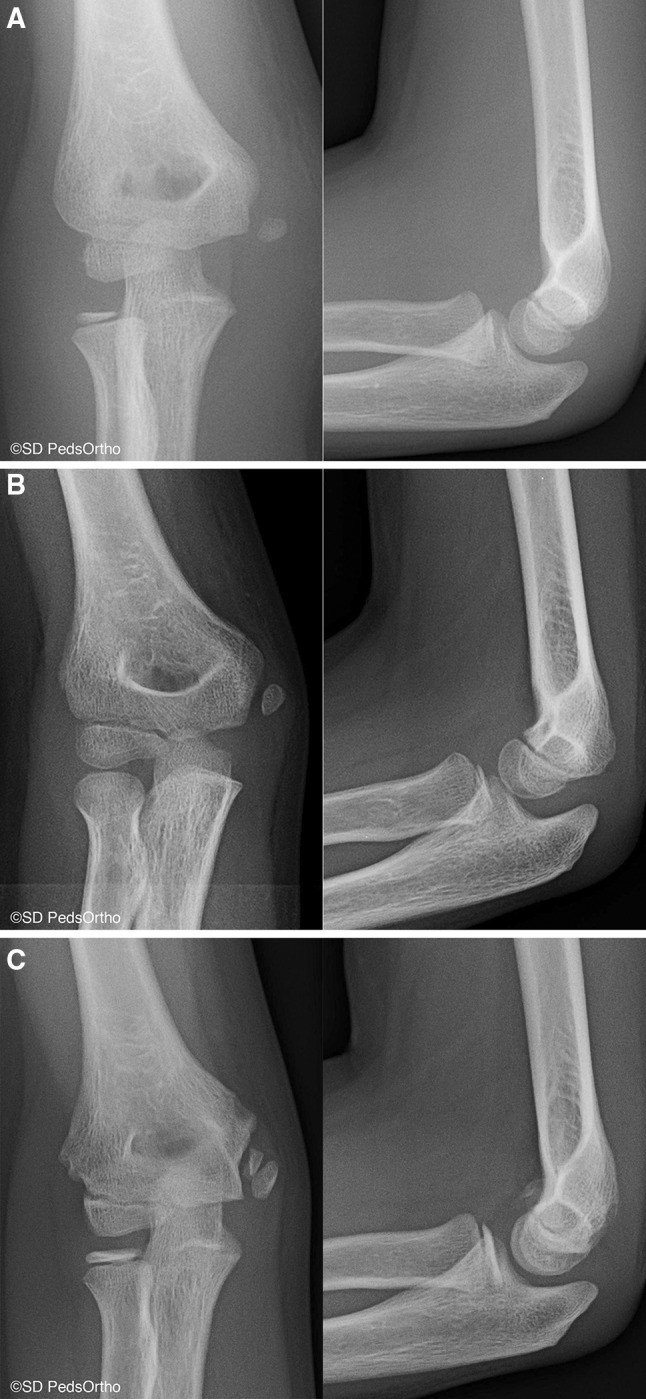
**a** Anteroposterior and lateral radiographs of an 8-year-old male revealing a displaced medial epicondyle fracture. **b** One month postinjury, the patient was pain-free. **c** At final follow-up (1.75 years postinjury), the patient had developed an asymptomatic osseous nonunion and had returned to full activities

**Fig. 3:**
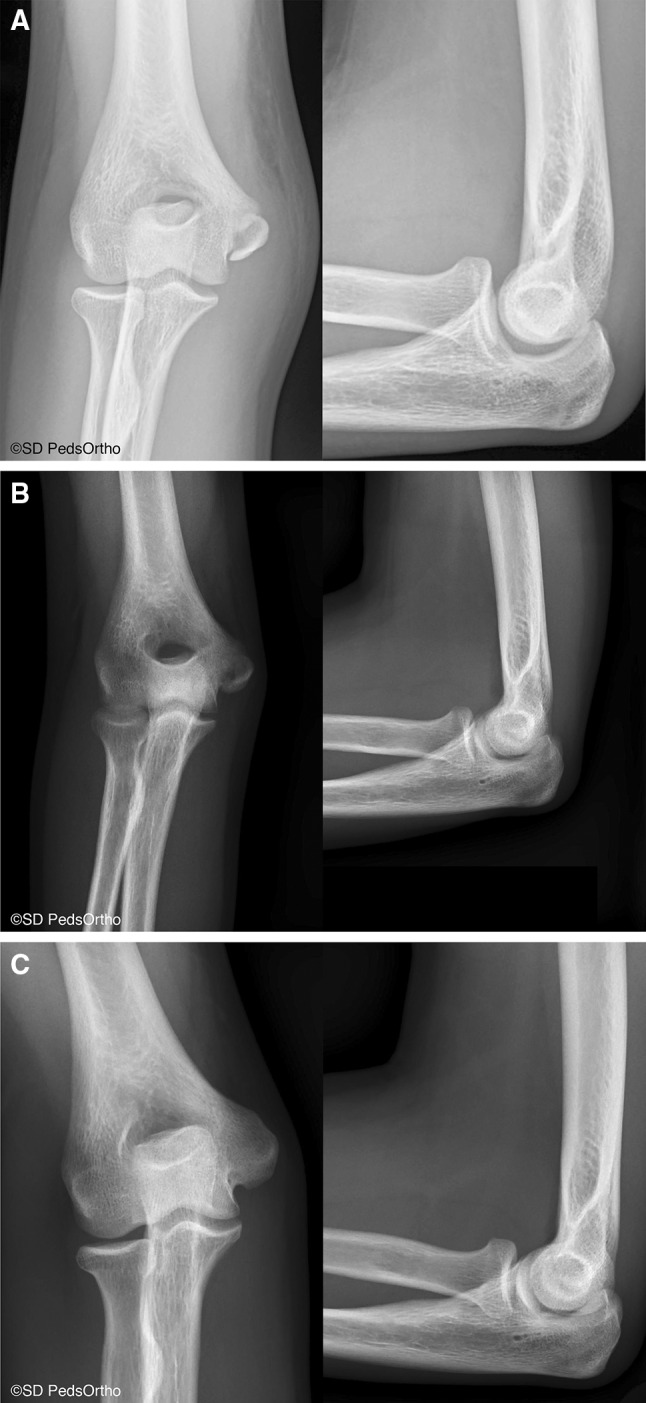
**a** Anteroposterior and lateral radiographs of a 15-year-old, right hand dominant, male, water polo player with an isolated right medial epicondyle fracture. **b** Two months postinjury, the fracture showed interval healing, and the patient was asymptomatic. **c** 1.5 years postinjury, the patient’s fracture had healed with a mild malunion that was asymptomatic and he returned to full sporting activities

To our knowledge, this is the first study that reports a direct comparison with prospective physical examination and validated outcome measures of cannulated screw fixation versus nonoperative treatment for isolated displaced medial humeral epicondyle fractures in children. Although our study sample is small, our results show that both operative and nonoperative management lead to high patient satisfaction and high outcome scores. As predicted, the rate of fracture nonunion is substantial (Fig. 2a–c), with only 33 % (2/6) of nonoperative patients having a documented union, one of which was malunited with increased medial epicondylar prominence compared to his contralateral elbow (Fig. 3a–c).

Farsetti et al., in 2001, reported on the long-term results of nonoperative management compared with K-wire/T-nail fixation or fragment excision with suture soft tissue reattachment [6]. While patients who underwent fragment excision had generally poor results, prompting them to recommend against this form of treatment, they also reported very good clinical results with nonoperative care and operative fixation. Similar to our study, the majority of nonoperative patients went on to radiographic osseous nonunion (17/19). All 17 operatively treated patients went on to bony union, but bony irregularities were always present. With screw fixation, we did not see the same irregularities in our study group, likely secondary to the increased fixation stability with compression screw versus. K-wire or T-nail constructs.

Interestingly, our operative patients, all with radiographic union, had a higher rate of tenderness to palpation than nonoperative patients (50 vs. 0 %). The fact that none of the nonoperative patients had tenderness on examination, even those with radiographic osseous nonunion, suggests that these patients went on to develop a mechanically stable and, therefore, asymptomatic, fibrous union. Stress radiographs could be utilized in future studies to confirm this theory. All of these were reported as mild with no associated pain at rest or limitations to sports. As all of our patients had healed surgical incisions from their ORIF in addition to retained screws, we suspect that the subcutaneous nature of the retained implant may lead to increased rates of point tenderness. Of note, no patient or family desired implant removal, despite the reported mild tenderness.

Good functional outcomes have also been reported in 2013 with operative and nonoperative treatment in young athletes by Lawrence et al. (6 nonoperative and 14 operative) [12]. While their final follow-up was limited to telephone interviews, they reported excellent DASH scores throughout, with high levels of patient satisfaction, independent of the treatment method. Their treatment algorithms lead their nonoperative patients to have less fracture displacement on average and a lower energy mechanism when compared to their operative group. This inherent dichotomy makes it difficult to make broad treatment generalizations for all patients presenting with isolated fractures. Unlike our study where two out of six nonoperative patients, both with osseous nonunion, were unable to return to the desired sport, they found that all nonoperative patients went onto bony union and were able to return to their sport after injury. This difference may be secondary to their lower average displacement than our population (5.3 vs. 9 mm), which also likely reflects a lower energy of the initial trauma.

In their article, “Medial epicondyle fractures in children: clinical decision making in the face of uncertainty”, Mehlman and Howard report succinctly on the difficulties surrounding the appropriate care of medial humeral epicondyle fractures [13]. While this study attempted to address some of the previously noted limitations that were made apparent in the article, it, too, has several limitations. While our data collection was prospective with an average of greater than 3 years of follow-up, all patients were identified retrospectively. Our inclusion and exclusion criteria attempted to identify similar groups of patients, though our lack of dislocations in the nonoperative group suggests that treatment bias did occur and our groups had inherent differences. It is our group’s belief that the standard immobilization used for nonoperative management would likely increase the rate of elbow stiffness when used in fractures associated with an elbow dislocation. Therefore, these patients are typically treated surgically to allow for earlier mobilization. In addition, as we relied on documented dislocation with formal reduction, the actual number of fractures associated with elbow dislocations in this study could be falsely low, given the occurrence of spontaneous reductions that were never recorded. The monetary reimbursement and the overall small percentage of patients available for analysis also highlight the high potential for selection bias as a further limitation. With less than 10 % of patients returning for evaluation from the identified 140 patients who met the inclusion criteria, our ability to make definitive recommendations or meaningful statistical analysis is also limited.

Finally, and perhaps most importantly, this study relies on imprecise methods to classify initial fracture displacement. Pappas et al. clearly showed that there was poor reliability in the determination of displacement when standard anteroposterior and lateral radiographs are utilized [4]. CT analysis has further demonstrated that true displacement can be significantly different to what is interpreted from standard radiographs [5]. The addition of advanced imaging with ultrasound, CT, magnetic resonance imaging (MRI), or standard use of the distal humerus axial view, as recently described by Souder et al. [14] to better evaluate and quantify medial humeral epicondyle displacement, would have added substantial value to the initial evaluation of these patients.

In this small cohort, operative management of displaced distal humerus medial epicondyle fractures resulted in a higher rate of fracture union and return to sports, despite a higher rate of medial epicondyle tenderness. Further research is required with carefully designed prospective studies utilizing accurate imaging techniques and strict randomization to determine if obtaining a surgical union via ORIF is clinically superior to accepting a higher rate of radiographic nonunion and malunion.
